# Detailed deletion mapping at chromosome 11q23 in colorectal carcinoma

**DOI:** 10.1054/bjoc.2000.1366

**Published:** 2000-08-17

**Authors:** A S-G Lee, Y-C Seo, A Chang, S Tohari, K-W Eu, F Seow-Choen, J O’D McGee

**Affiliations:** Department of Clinical Research, Department of Colorectal Surgery, Singapore General Hospital, Outram Road, 169608, Republic of Singapore; Nuffield Department of Medicine, John Radcliffe Hospital, Oxford, OX3 9DU, UK; Division of Medical Sciences, National Cancer Centre, 169610, Republic of Singapore

**Keywords:** loss of heterozygosity (LOH), chromosome 11q, tumour suppressor genes, colorectal carcinoma

## Abstract

Loss of heterozygosity (LOH) is frequent at the chromosomal region 11q22–q23 in several types of tumours of diverse cell origin. Previous investigations of LOH at this chromosomal region in colorectal carcinoma have been contradictory in their findings, and have only included between 1–4 loci. In order to define any regions of LOH on 11q23, we investigated 16 loci between D11S940 and D11S934 on the long arm of chromosome 11 using microsatellite analysis. Of 57 colorectal carcinomas specimens, 36 (63.2%) demonstrated LOH at one or more marker, with the highest frequencies of LOH at D11S1340 (41.0%), located between 105.13–111.97 Mb from the centromere, and D11S924 (37.1%) and D11S4107 (40.5%), both located approximately 113 Mb from the centromere. No statistically significant associations between LOH and age-of-presentation or Dukes’ stage were found. LOH was observed in colorectal tumours of all Dukes’ stages, including Dukes’ stages A and B, suggesting that the inactivation of a tumour suppressor gene(s) on 11q23 occurs in the early stages of colorectal carcinoma. These results confirm the presence of putative tumour suppressor gene(s) at chromosome 11q23, involved in the carcinogenesis of colorectal carcinoma, and will facilitate future identification of candidate genes. © 2000 Cancer Research Campaign


*British Journal of Cancer* (2000) **83**(6), 750–755
© 2000 Cancer Research Campaign
doi: 10.1054/ bjoc.2000.1366, available online at http://www.idealibrary.com on IDEAL®

### Detailed deletion mapping at chromosome 11q23 in colorectal carcinoma

**AS-G Lee[1,4], Y-C Seo[1], A Chang[1], S Tohari[1], K-W Eu[2], F Seow-Choen[2] and JO'D McGee[3]**

[1]Department of Clinical Research; [2]Department of Colorectal Surgery, Singapore General Hospital, Outram Road, Republic of Singapore 169608; [3]Nuffield Department of Medicine, John Radcliffe Hospital, Oxford OX3 9DU, UK; [4]Division of Medical Sciences, National Cancer Centre, Republic of Singapore 169610

**Summary** Loss of heterozygosity (LOH) is frequent at the chromosomal region 11q22–q23 in several types of tumours of diverse cell origin. Previous investigations of LOH at this chromosomal region in colorectal carcinoma have been contradictory in their findings, and have only included between 1–4 loci. In order to define any regions of LOH on 11q23, we investigated 16 loci between D11S940 and D11S934 on the long arm of chromosome 11 using microsatellite analysis. Of 57 colorectal carcinomas specimens, 36 (63.2%) demonstrated LOH at one or more marker, with the highest frequencies of LOH at D11S1340 (41.0%), located between 105.13–111.97 Mb from the centromere, and D11S924 (37.1%) and D11S4107 (40.5%), both located approximately 113 Mb from the centromere. No statistically significant associations between LOH and age-of-presentation or Dukes' stage were found. LOH was observed in colorectal tumours of all Dukes' stages, including Dukes' stages A and B, suggesting that the inactivation of a tumour suppressor gene(s) on 11q23 occurs in the early stages of colorectal carcinoma. These results confirm the presence of putative tumour suppressor gene(s) at chromosome 11q23, involved in the carcinogenesis of colorectal carcinoma, and will facilitate future identification of candidate genes. © 2000 Cancer Research Campaign

**Keywords**: loss of heterozygosity (LOH); chromosome 11q; tumour suppressor genes; colorectal carcinoma

Colorectal carcinoma is one of the leading causes of cancer mortality in the Western world, with an increasing incidence in Singapore (Chia et al, 1996; Wingo et al, 1998). The development of colorectal carcinoma is a multi-step progression with transformation of the normal colonic epithelium to an adenomatous polyp and ultimately an invasive cancer (Gryfe et al, 1997). Genetic alterations that are involved during this progression include the dysregulation of the K-ras gene and LOH of chromosomes 5q, 17p and 18q. These chromosomal regions harbour several tumour suppressor genes: the adenomatous polyposis gene (APC)(5q), TP53 gene (17p), and the deleted in colorectal cancer (DCC) gene (18q) (Fearon and Vogelstein, 1990; Gryfe et al, 1997).

Recent independent analyses of chromosome 11q suggest that a putative tumour suppressor gene or genes located in chromosome 11q22–q24 may be involved in the tumorigenesis of several solid tumours of diverse cell types, such as tumours of the breast (Carter et al, 1994; Negrini et al, 1995; Tomlinson et al, 1995), ovary (Davis et al, 1996; Gabra et al, 1996), stomach (Baffa et al, 1996), lung (Rasio et al, 1995), cervix (Hampton et al, 1994), nasopharynx (Hui et al, 1996) and malignant melanoma (Tomlinson et al, 1993; Herbst et al, 1995; Robertson et al, 1996). The involvement of this chromosomal region in the tumorigenesis of colorectal cancer is unclear, with cytogenetic (Konstantinova et al, 1991; Keldysh et al, 1993) and some molecular studies (Tomlinson and Bodmer, 1996; Connolly et al, 1999) indicating losses in this region but with other molecular studies reporting no significant losses (Koreth et al, 1997). These studies employed four or fewer loci from the 11q22–23 region, an insufficient number to establish clearly the regions of 11q deletion in colorectal carcinogenesis.

In order to confirm whether the chromosomal region 11q22–23 is indeed lost in colorectal carcinoma and also to identify the regions of loss, we have performed a comprehensive genetic analysis of chromosome 11q22–q23 in 57 colorectal tumours using 16 highly polymorphic microsatellite markers. We have demonstrated that loss of heterozygosity at this chromosomal region is common in colorectal carcinoma. The critical regions of LOH defined by this study will be crucial for future work on the identification of putative tumour suppressor gene(s) by positional cloning techniques. We have also observed LOH on 11q23 in tumours from patients with early Dukes' Stage (A and B), implying that loss of 11q is an early event in colorectal cancer.

### MATERIALS AND METHODS

### Samples

Fifty-seven colorectal carcinoma samples and corresponding normal colonic mucosa were snap-frozen in liquid nitrogen upon resection and stored at –70°C. Peripheral blood samples were collected from each of the patients in EDTA tubes and stored at –70°C. Fifty-five cases were identified histopathologically as adenocarcinoma, and two cases were mucinous adenocarcinoma. Four (7.1%) of the patients presented at Dukes' stage A, 15 (26.8%) were Stage B, 17 (30.4%) were Stage C and 20 (35.7%) were stage D. The Dukes' Stage for one patient was unknown.

### DNA extraction

Cryostat sections were cut from each of the tumours, stained with H&E and viewed. The tumours were then trimmed to exclude

*Received 6 December 1999*
*Revised 11 May 2000*
*Accepted 8 June 2000*

*Correspondence to:* A S-G Lee normal cells and for the enrichment of neoplastic cells. Cryostat sections of normal colonic mucosa were also viewed to confirm the exclusion of neoplastic cells. DNA was extracted from tissues using DNAzol (GIBCO/BRL, USA) according to the manufacturer's instructions. DNA was extracted from frozen whole blood using sucrose lysis buffer and proteinase K digestion. The extracted DNA was quantified by spectrophotometry.

### Microsatellite analysis

Sixteen microsatellite markers from the chromosomal region 11q22–23 were selected: D11S940, D11S1778, D11S1818, D11S1340, D11S29, D11S1356, D11S4104, D11S924, D11S4171, D11S4132, D11S925, D11S4107, D11S1345, D11S1328, D11S933 and D11S934. Two microsatellite markers from chromosome 11p, D11S929 and D11S1344 were selected as controls. The primer sequences, chromosome localization and frequency of heterozygosity were obtained electronically from the Genome Database and the US National Center for Biotechnology Information (NCBI) database. Polymerase chain reactions (PCR) were performed in a 10 μl volume with 100–400 ng of DNA, 125 μM of each dNTP, 0.5 μM of each primer, 1.5 mM $MgCl_2$ and 0.2 units of *Taq* DNA polymerase (Promega, USA). The sense primer was end-labelled with [$\gamma$-$^{33}$P]dATP (Amersham, USA or NEN, USA). The PCR conditions were an initial denaturation step at 94°C for 3 min, 25 cycles of denaturation (1 min at 94°C), annealing (1 min at 60–67°C), and extension (1 min at 72°C), and a final extension step (72°C, 7–15 min). Annealing temperatures were optimized for each primer pair. The PCR products were separated on an 8% polyacrylamide sequencing gel and exposed to X-ray film overnight and also exposed to the CS phosphor screens (Biorad, USA) for 4–6 h.

### Assessment of LOH

DNA quantitation was performed by scanning the exposed CS phosphor screens with the Molecular Imager (Model GS-250, Biorad, USA). LOH was determined by quantitation of the signal intensity of each allele, and comparing the ratios of the intensity of the alleles from the tumour DNA with that of the constitutional (normal mucosa or blood) DNA. The difference in allele ratios between normal and tumour samples was divided by the allele ratio for the normal sample and a value of over 0.3 was scored as LOH, as has been previously described by other investigators studying LOH in this region (Hampton et al, 1994; Negrini et al, 1995; Connolly et al, 1999).

Calculation of allele ratios were determined by the allelic ratio method as well (Cawkwell et al, 1993), to allow for the comparison of our data with that of Koreth et al (1997). Briefly, the ratio of alleles was calculated for each normal and tumour sample and then the tumour ratio was divided by the normal ratio, i.e. T1:T2/N1:N2, and with a cut-off ratio of 1.5. All samples with LOH were repeated to confirm the result.

### DNA fingerprinting

All samples with microsatellite instability (MI) were subjected to DNA fingerprinting using the D1S80 microsatellite marker in order to verify that the normal and tumour samples were from the same patient and not from unrelated patients. The extracted DNA

**Table 1** The frequency of LOH at 16 microsatellite markers on chromosome 11q22–q23 and two microsatellite markers on chromosome 11p in colorectal carcinoma

| Marker | Distance from centromere in megabases (min/max)[a] | No. with LOH/No. of informative cases (%LOH)[b] | No. with LOH/No. of informative cases (%LOH)[c] |
|---|---|---|---|
| D11S929 | 19.38/19.79[d] | 4/33 (12.1) | 1/33 (3.0) |
| D11S1344 | 35.07/53.57[d] | 8/32 (25.0) | 5/32 (15.6) |
| D11S940 | 91.97/97.42 | 9/41 (22.0) | 6/41 (14.6) |
| D11S1778 | 101.56/105.43 | 12/44 (27.3) | 11/44 (25.0) |
| D11S1818 | 104.23/110.40 | 16/46 (34.8) | 15/46 (32.6) |
| D11S1340 | 105.13/111.97 | 16/39 (41.0) | 14/39 (35.9) |
| D11S29 | 110.64/120.93 | 15/46 (32.6) | 14/46 (30.4) |
| D11S1356 | 106.59/108.87, 111.30/111.97 | 14/46 (30.4) | 14/46 (30.4) |
| D11S4104 | 112.75/112.75 | 9/38 (23.7) | 7/38 (18.4) |
| D11S924 | 113.02/113.02 | 13/35 (37.1) | 12/35 (34.3) |
| D11S4171 | 113.14/113.14 | 13/41 (31.7) | 13/41 (31.7) |
| D11S4132 | 113.28/113.28 | 7/37 (18.9) | 6/37 (16.2) |
| D11S925 | 113.41/115.23, 115.62/115.62 | 14/43 (32.6) | 13/43 (30.2) |
| D11S4107 | 113.54/113.60 | 17/42 (40.5) | 15/42 (35.7) |
| D11S1345 | 117.40/117/40, 118.32/118.60 | 6/43 (14.0) | 5/43 (11.6) |
| D11S1328 | 118.92/119.09, 119.58/119.58 | 9/41 (22.0) | 8/41 (19.5) |
| D11S933 | 119.19/121.31 | 7/27 (25.9) | 6/27 (22.2) |
| D11S934 | 120.21/121.31 | 11/33 (33.3) | 10/33 (30.3) |

[a]The distance from the centromere was obtained from the Genome Database (http//:gdbwww.gdb.org); [b]Assessment of LOH using the 30% cutoff as previously described (Hampton et al, 1994; Negrini et al, 1995; Koreth et al, 1997); [c]Assessment of LOH using the 50% cutoff as previously described (Cawkwell et al, 1993; Connolly et al, 1999); [d]Distance from the centromere on chromosome 11p

© 2000 Cancer Research Campaign 

| | Colorectal carcinoma cases | | | | | | | | | | | | | | | | | | | | | | | | | |
|---|---|---|---|---|---|---|---|---|---|---|---|---|---|---|---|---|---|---|---|---|---|---|---|---|---|---|
| **Marker** | 54 | 66 | 67 | 68 | 71 | 1 | 2 | 6 | 20 | 53 | 18 | 27 | 28 | 29 | 42 | 39 | 40 | 51 | 58 | 25 | 50 | 5 | 23 | 36 | 64 | 14 |
| D11S940 | he | he | ho | he | he | he | he | he | he | ho | ho | he | ho | he | ho | he | ho | ho | he | he | he | he | he | L | he | ho |
| D11S1778 | he | he | he | ho | ho | he | ho | he | he | he | he | he | ho | he | he | he | he | he | he | he | he | ho | L | he | he | L |
| D11S1818 | he | ho | he | he | he | ho | he | he | he | he | he | he | he | he | he | he | he | he | he | he | ho | he | he | he | he | L |
| D11S1340 | he | ho | he | he | he | ho | he | ho | he | ho | he | he | ho | he | ho | he | ho | ho | he | he | he | he | he | he | L | ho |
| D11S29 | he | he | he | he | he | he | he | he | he | he | he | nd | he | he | he | ho | ho | he | ho | he | he | he | he | ho | he | he |
| D11S1356 | ho | he | he | he | ho | he | ho | ho | he | he | ho | he | he | he | he | he | he | he | he | he | he | he | he | ho | he | he |
| D11S4104 | ho | he | he | he | he | he | he | he | he | he | he | he | ho | ho | he | ho | he | he | he | he | he | he | he | he | ho | he |
| D11S924 | ho | ho | he | he | ho | ho | ho | ho | ho | ho | ho | he | he | ho | he | he | he | he | he | he | he | ho | ho | he | he | he |
| D11S4171 | ho | he | he | ho | he | he | he | he | ho | he | he | ho | nd | he | he | he | ho | he | he | he | he | L | he | he | L | he |
| D11S4132 | he | he | he | he | ho | ho | ho | he | he | he | he | he | he | ho | ho | he | ho | he | he | he | he | he | he | he | he | he |
| D11S925 | he | he | ho | he | he | ho | he | he | he | he | he | ho | he | he | ho | he | he | ho | he | he | L | ho | he | he | he | he |
| D11S4107 | he | he | he | he | he | he | he | ho | ho | he | ho | nd | nd | he | he | he | ho | he | L | L | he | he | he | he | he | ho |
| D11S1345 | he | ho | he | ho | ho | he | ho | he | he | he | ho | he | he | he | ho | he | he | he | he | he | he | he | he | he | ho | he |
| D11S1328 | ho | he | he | he | ho | he | ho | he | he | he | he | ho | he | he | he | he | he | he | he | he | he | he | ho | he | he | ho |
| D11S933 | ho | he | he | he | ho | ho | he | he | ho | he | he | he | ho | ho | ho | he | he | he | he | ho | he | he | ho | ho | he | he |
| D11S934 | he | ho | he | he | ho | ho | he | he | ho | ho | ho | he | he | he | ho | ho | he | ho | he | ho | he | he | ho | he | he | ho |
| Dukes' Stage | B | D | D | D | A | B | A | D | C | B | D | A | C | B | D | C | D | C | D | C | D | B | B | C | C | B |

| | Colorectal carcinoma cases | | | | | | | | | | | | | | | | | | | | | | | | | | | | | | |
|---|---|---|---|---|---|---|---|---|---|---|---|---|---|---|---|---|---|---|---|---|---|---|---|---|---|---|---|---|---|---|---|
| **Marker** | 21 | 38 | 48 | 34 | 16 | 44 | 57 | 31 | 55 | 63 | 30 | 60 | 8 | 59 | 10 | 11 | 33 | 47 | 15 | 35 | 69 | 12 | 4 | 49 | 70 | 24 | 62 | 65 | 45 | 43 | 46 |
| D11S940 | he | L | he | L | he | he | he | he | ho | ho | he | L | he | L | L | L | ho | L | L | he | M | M | ho | he | M | he | ho | he | he | he | he |
| D11S1778 | he | ho | L | L | he | he | he | he | ho | L | he | L | L | ho | he | ho | L | L | he | he | M | he | M | he | L | L | L | he | M | he | ho |
| D11S1818 | he | he | ho | ho | L | L | he | L | L | L | he | ho | L | L | L | L | L | L | L | L | M | M | M | M | L | L | M | he | he | he | he |
| D11S1340 | he | ho | L | ho | L | ho | L | ho | L | he | L | L | L | he | L | L | L | ho | L | L | M | M | L | L | L | he | he | ho | he | ho | he |
| D11S29 | he | ho | he | L | L | ho | L | he | L | he | he | L | L | ho | L | L | L | L | L | L | M | L | L | he | L | ho | he | he | he | ho | he |
| D11S1356 | L | he | he | he | he | he | ho | L | ho | he | L | L | L | L | L | he | L | L | L | L | M | M | he | ho | L | L | L | he | he | he | he |
| D11S4104 | he | ho | he | ho | ho | he | L | L | ho | L | L | ho | ho | L | L | he | ho | ho | L | L | M | M | ho | he | L | he | ho | he | M | he | ho |
| D11S924 | L | he | ho | ho | ho | ho | ho | he | L | L | ho | L | L | he | L | L | L | L | L | L | L | L | ho | he | ho | he | ho | he | he | he | ho |
| D11S4171 | ho | ho | he | ho | he | he | L | he | L | L | L | L | ho | L | ho | he | L | L | L | L | M | L | ho | he | ho | ho | he | he | M | he | he |
| D11S4132 | ho | he | ho | ho | ho | he | he | he | L | ho | L | ho | ho | L | ho | L | ho | ho | L | L | M | M | he | he | ho | L | he | he | M | he | he |
| D11S925 | he | he | L | he | L | L | L | he | ho | he | ho | ho | L | L | ho | L | ho | L | L | L | L | M | he | ho | L | L | he | M | he | he | he |
| D11S4107 | he | L | he | ho | ho | L | L | he | L | L | ho | L | L | L | L | L | L | L | L | L | M | M | M | he | L | he | he | he | ho | he | M |
| D11S1345 | he | he | he | ho | L | he | he | he | ho | he | ho | he | L | he | L | he | L | L | ho | ho | M | he | he | he | L | he | he | he | he | he | ho |
| D11S1328 | ho | he | he | ho | he | he | ho | L | ho | he | L | L | he | L | L | L | ho | ho | L | L | M | M | he | he | L | M | he | he | he | ho | he |
| D11S933 | ho | ho | ho | ho | ho | L | he | L | ho | L | L | ho | ho | ho | ho | ho | L | ho | ho | ho | L | M | he | he | L | ho | ho | he | M | M | ho |
| D11S934 | ho | ho | he | L | he | L | ho | L | ho | ho | he | ho | ho | L | ho | L | L | L | ho | L | L | M | he | M | L | he | ho | L | he | he | he |
| Dukes' Stage | C | C | C | D | D | D | C | n.d | C | D | D | C | C | C | B | B | A | B | D | D | C | B | B | B | D | B | C | D | B | D | D |

was amplified by PCR, as described above, except that end-labelling with [γ-[33]P]dATP was not done. The PCR products were run on a 3% Metaphor (FMC, USA) agarose gel to determine the sizes of the products.

### Statistical analysis

The Fisher's exact test and Chi-squared test were used to determine any associations between LOH and clinicopathological parameters. Information on Dukes' stage was available for 56 of the 57 patients.

### RESULTS

Table 1 shows the frequency of loss of heterozygosity (LOH) in the 57 colorectal carcinomas. The results of microsatellite analyses for all samples are shown in Table 2. Table 3 shows the correlation of clinicopathological parameters and presence or absence of LOH. Representative examples of LOH and microsatellite instability (MI) are shown in Figures 1 and 2 respectively.

To determine the prevalence of LOH and MI on chromosome 11q22–q23 in colorectal carcinoma, 16 highly polymorphic microsatellite markers spanning this region were utilized to compare the tumour DNA and corresponding normal DNA from 57 colorectal carcinoma patients. Enrichment of neoplastic cells was achieved by the cryosectioning technique in order to reduce the proportion of contaminating non-neoplastic cells which would reduce or mask the observation of LOH. Accurate assessment of LOH was derived from direct measurement of signal intensities of each allele using phosphor screens and a molecular imager.

This study has defined three markers with frequent LOH, D11S1340 (41.0%), D11S924 (37.1%) and D11S4107 (40.5%), using the 30% cutoff value (Table 1). D11S1340 is located 105.13–111.97 Mb from the centromere. Both D11S924 and D11S4107 are located approximately 113 Mb from the centromere on the long arm of chromosome 11 (Table 1). The lowest frequency of LOH at the 11q23 region was at D11S1345 with a frequency of 14.0% (Table 1). The frequency of LOH on the short arm of chromosome 11 was 12.1% at D11S929 (11p14), and 25% at D11S1344 (11p11–12).

Overall, 36 of the 57 colorectal cases (63.2%) showed LOH for at least one marker on chromosome 11q22–q23 (Table 2). LOH was calculated using 0.3 as the cutoff value (Hampton et al, 1994; Negrini et al, 1995; Connolly et al, 1999). A complex pattern of LOH was observed in this series of tumours, with regions exhibiting allelic losses interspersed with regions where heterozygosity was retained (Table 2), and this is likely to be caused by the different underlying genetic mechanism of allele loss. Three cases, 55, 33 and 47, showed probable chromosomal non-disjunction, consistent with loss of the entire chromosome 11, with either LOH or homozygosity observed at all microsatellite markers (Table 2). Terminal deletion was observed in eleven cases (34, 44, 31, 59, 11, 33, 47, 35, 69, 70, and 65) (Table 2). Interstitial allele loss caused by mitotic recombination, deletion or gene conversion was observed in the majority of the remaining cases with LOH and have become apparent in this study due to the large number of microsatellite markers used to examine the frequency of LOH.

Microsatellite instability (MI) is depicted by numerous or aberrant alleles at a microsatellite locus indicating genomic instability. Microsatellite instability was detected in 11 (19.3%) of 57 colorectal carcinoma samples and was detected at all the microsatellite markers except for D11S924 (Table 2). This frequency of MI is similar to that of other studies of MI in sporadic colorectal cancer (Risio et al, 1996; Gryfe et al, 1997). Six tumours displayed MI at only one of the markers and five tumours had MI at two or more markers, suggesting the involvement of mismatch repair genes in the development of these tumours (Table 2). Representative examples of MI are shown in Figure 2. DNA fingerprinting with the D1S80 marker was done on all these 11 cases with microsatellite instability and showed that the normal and tumour DNA for these patients were matched and not from different individuals.

No associations were seen between the presence of LOH and Dukes' stage or the patient's age at diagnosis (Table 3). LOH was detected in one of four (25.0%) cases at Dukes' stage A, 10 of 15 (66.7%) cases at stage B, 13 of 17 cases at stage C (76.5%) and 10 of 20 (50.0%) cases at stage D (Table 3).

### DISCUSSION

This is the first paper describing a detailed deletion mapping of the chromosome 11q22–q23 region in colorectal carcinoma, utilizing 16 microsatellite markers to this region. The highest frequencies of LOH were observed at D11S1340 (41.0%), located 105.13–111.97 Mb from the centromere, and at D11S924 (37.1%) and D11S4107 (40.5%), both located approximately 113 Mb from the centromere. These two regions were bordered by microsatellite markers showing lower frequencies of LOH. All markers (D11S924, D11S4171, D11S925, D11S4107) located approximately 113 Mb from the centromere had frequencies of LOH above 30% with the exception of one marker, D11S4132, which could possibly be due to inaccurate chromosomal localization. This study has further refined the region of LOH on 11q23, with our data suggesting the possibility of two defined regions of loss, rather than one region

 © 2000 Cancer Research Campaign

**Table 3** LOH and clinicopathological parameters

| | LOH (%) | No LOH (%) | Statistical test | *P* |
|---|---|---|---|---|
| Age at presentation | | | Fisher's exact test | 0.163 |
| ≤ 50 years | 10 (79.9) | 3 (23.1) | | |
| > 50 years | 25 (56.8) | 19 (43.2) | | |
| Dukes' Stage | | | Chi-squared test by exact method | 0.156 |
| A | 1 (25.0) | 3 (75.0) | | |
| B | 10 (66.7) | 5 (33.3) | | |
| C | 13 (76.5) | 4 (23.5) | | |
| D | 10 (50.0) | 10 (50.0) | | |

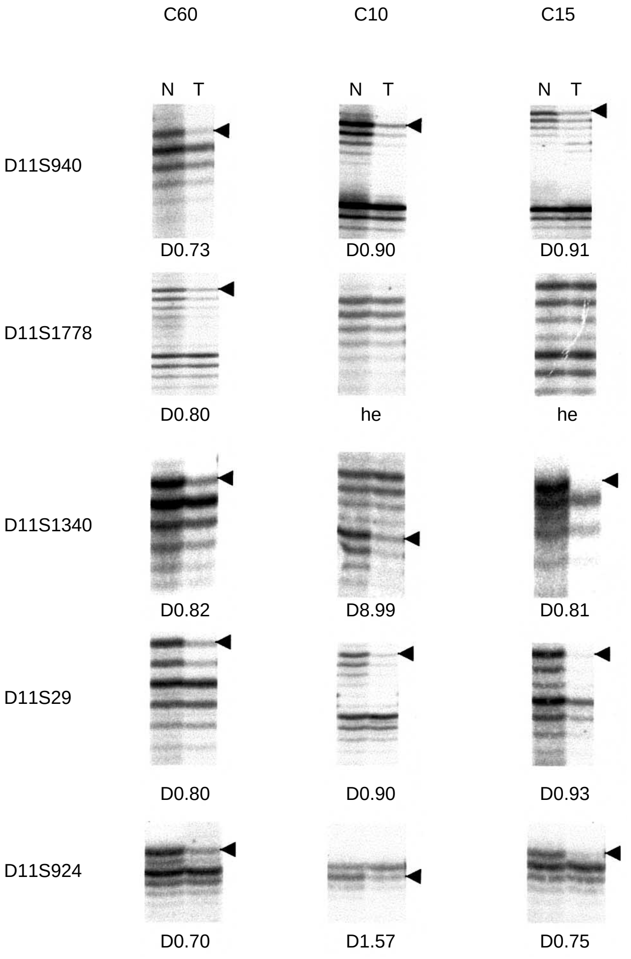

**Figure 1** Examples of LOH on chromosome 11 in representative colorectal carcinoma samples. *Top* = case numbers; *Left* = microsatellite markers listed from the most centromeric to the most telomeric; *N* = normal; *T* = tumour. Shown at the *bottom* of each autoradiograph is D, the difference in allele ratios between normal and tumour samples, divided by the allele ratio for the normal sample. When the allele ratio in tumour DNA differed more than 30% from the ratio of alleles in normal DNA, LOH was scored. *Arrowheads* indicate the allele lost in tumour DNA

spanning 4.9 Mb, between D11S897 and D11S925 as reported by Connolly et al (1999).

Previous RFLP and microsatellite analyses of the chromosomal region 11q22–q23 in colorectal carcinoma have only utilized between one to four loci, with conflicting data on the frequency of loss of heterozygosity (LOH). Koreth et al (1997) reported a low frequency of LOH of 11–12% in this area, utilizing two microsatellite markers to this region, D11S35 and D11S29. Moderate levels of LOH were obtained at the D11S29 locus (29%) and the dopamine D2 receptor locus (33.8%) in two other studies (Gustafson et al, 1994; Tomlinson and Bodmer, 1996). By employing RFLP methods and studying only four loci at this chromosomal region, Keldysh et al (1993) detected LOH at a high frequency of 50–60% in the same chromosomal region. Recently, high frequencies of LOH of 40% and 47.6% were reported by Connolly et al (1999), in their study employing two microsatellite markers in this region, D11S897 and D11S925. Our findings of high frequencies of LOH provides further evidence that the chromosomal region 11q23 is indeed lost during colorectal carcinogenesis.

Variations in the frequencies of LOH between studies of the same tumour type and using the same microsatellite marker may be due to the different cutoff criteria used for the assessment of LOH. In order to compare our microsatellite analysis with those from other studies, we have reported the LOH frequency using the 30% cutoff value (Hampton et al, 1994; Negrini et al, 1995; Connolly et al, 1999) and the 50% cutoff value (Cawkwell et al, 1993; Koreth et al, 1997) (Table 1). We note a general reduction of %LOH using the 50% cutoff criteria, described by Cawkwell et al (1993). Using the 30% cutoff, LOH at D11S925 was observed in 47.6% of cases analysed by Connolly et al (1999) but in only 32.6% of our cases. Nevertheless, these values are higher than that reported by Koreth et al (1997) at D11S29 of 12%, which was assessed using the 50% cutoff. A similar analysis of this same marker, D11S29, in our case showed LOH of 30.4%. However, we conclude that either criteria may be used to define the frequencies of LOH within a study, as both criteria allow the identification of high LOH rates in relation to LOH rates observed at other markers in that study. The highest rates of LOH in this study, at D11S1340, D11S924 and D11S4107, were observed at the same markers, for both cutoff values. Reporting of LOH frequency using both criteria may be helpful for comparisons between studies.

By utilizing an extensive panel of microsatellite markers in this study, we have excluded the possibility of these losses being a reflection of generalized chromosomal instability. The low frequency of LOH on the short arm of chromosome 11 at D11S929 was similar in frequency to that at D11S1345, on 11q. In contrast, higher frequencies of LOH were observed at the D11S1340, D11S924 and D11S4107 on 11q23.

The D11S1340 locus is located approximately 105.13–111.97 Mb from the centromere. Also located in this region is the recently identified putative tumour suppressor gene, PPP2R1B, which encodes the β isoform of the A subunit of the serine/threonine protein phosphatase (PP2A). PP2A has been linked to

*© 2000 Cancer Research Campaign*

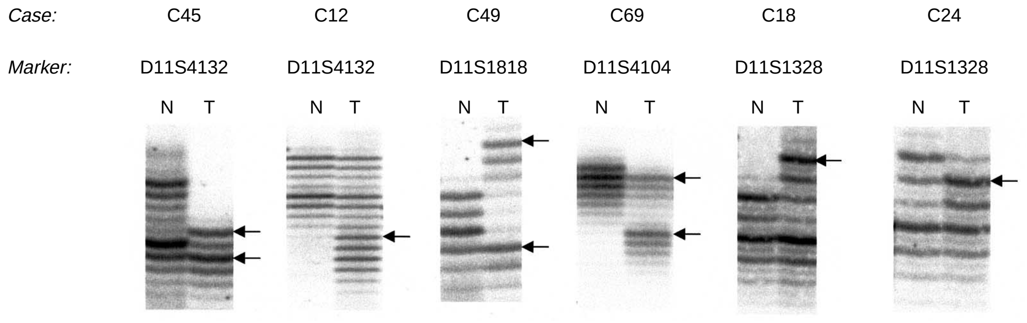

**Figure 2** Examples of microsatellite instability (MI) in representative colorectal carcinoma samples. *Top* = case numbers and microsatellite markers; *N* = normal; *T* = tumour. MI as depicted by additional bands in the tumour DNA are shown arrowed carcinogenesis, is involved in the down-regulation of the mitogen-activated protein kinase cascade and relays signals for cell proliferation. Alterations of the PPP2R1B gene were detected in human lung and colon cancer (Wang et al, 1998). This gene was identified from a region exhibiting high frequency of LOH of 42.9% and 46.2% respectively, using the D11S1647 and D11S1987 loci (Wang et al, 1998).

Both the loci D11S924 and D11S4107 are located approximately 113 Mb from the centromere. Deletions in this region have been detected in tumours of the nasopharynx (Hui et al, 1996), cervix (Hampton et al, 1994), ovary (Gabra et al, 1996), breast (Negrini et al, 1995) and lung (Rasio et al, 1995). Other genes located in this region include the CBL gene which participates in the signal transduction of haematopoietic cells (Blake et al, 1991) and the MLL gene which is translocated in acute leukaemias and possibly acts as a transcriptional regulatory factor (Gu et al, 1994). Other possible candidate genes include the LOH11CR2A gene that has been molecularly cloned (Monaco et al, 1997) and the BRCA3 gene which has been associated with the constitutional translocation 11q;22q (Iselius et al, 1983; Lindblom et al, 1994).

We have observed LOH on 11q23 in colorectal tumours from patients with Dukes' stage A (25%) and B (67%), suggesting that loss of function of a tumour suppressor gene on 11q23 occurs in the early stages of colorectal cancer. This finding is consistent with those of Evans et al (1998) and Davis et al (1996) who have reported that 11q deletion occurs early in cervical and ovarian neoplasia.

Our study has confirmed that LOH on chromosome 11q23 plays an important role in the pathogenesis of colorectal cancer. The two regions of deletion defined here suggest the presence of at least two putative tumour suppressor genes on 11q23 and will facilitate the identification of candidate tumour suppressor genes from this region.

### ACKNOWLEDGEMENTS

This work was supported by grants from the National Medical Research Council (NMRC) of Singapore, The Singapore Cancer Society and the Singapore General Hospital (SGH) Research Fund. We thank Ms Stephanie Fook Chong for statistical assistance, Dr PY Cheah for critical reading of the manuscript and A/Prof KC Soo for use of laboratory facilities at the Department of General Surgery, Singapore General Hospital.

### REFERENCES

Baffa R, Negrini M, Mandes B, Rugge M, Ranzani GN, Hirohashi S and Croce CM (1996) Loss of heterozygosity of chromosome 11 in adenocarcinoma of the stomach. *Cancer Res* **56**: 268–272

Blake TJ, Shapiro M, Morse HC III and Langdon WY (1991) The sequences of the human and mouse c-cbl proto-oncogenes show v-cbl was generated by a large truncation encompassing a proline-rich domain and a leucine zipper-like motif. *Oncogene* **6**: 653–657

Carter SL, Negrini M, Baffa R, Gillum DR, Rosenberg AL, Schwartz GF and Croce CM (1994) Loss of heterozygosity at 11q22–q23 in breast cancer. *Cancer Res* **54**: 6270–6274

Cawkwell L, Bell SM, Lewis FA, Dixon MF, Taylor GR and Quirke P (1993) Rapid detection of allele loss in colorectal tumours using microsatellites and fluorescent DNA technology. *Br J Cancer* **67**: 1262–1267.

Chia KS, Lee HP, Seow A and Shanmugaratnam K (1996) Trends in cancer incidence in Singapore 1968–1992. In *Singapore Cancer Registry Report No. 4*, pp. 13–15. Singapore Cancer Registry

Connolly KC, Gabra H, Millwater CJ, Taylor KJ, Rabiasz GJ, Watson JEV, Smyth JF, Wyllie AH and Jodrell DI (1999) Identification of a region of frequent loss of heterozygosity at 11q24 in colorectal cancer. *Cancer Res* **59**: 2806–2809

Davis M, Hitchcock A, Foulkes WD and Campbell IG (1996) Refinement of two chromosome 11q regions of loss of heterozygosity in ovarian cancer. *Cancer Res* **56:** 741–744

Evans MF, Koreth J, Bakkenist CJ, Herrington CS and McGee JO'D (1998) Allelic deletion at 11q23.3-q25 is an early event in cervical neoplasia. *Oncogene* **16**: 2557–2564

Fearon ER and Vogelstein B (1990) A genetic model for colorectal tumorigenesis. *Cell* **61:** 759–767

Gabra H, Watson JEV, Taylor KJ, Mackay J, Leonard RCF, Steel CM, Porteous DJ and Smyth JF (1996) Definition and refinement of a region of loss of heterozygosity at 11q23.3–q24.3 in epithelial ovarian cancer associated with poor prognosis. *Cancer Res* **56**: 950–954

Gryfe R, Swallow C, Bapat B, Redston M, Gallinger S and Couture J (1997) Molecular biology of colorectal cancer. *Curr Probl Cancer* **21:** 233–300

Gu Y, Alder H, Nakamura T, Schichman SA, Pradsad R, Canaani O, Saito H, Croce CM and Canaani E (1994) Sequence analysis of the breakpoint cluster region in the ALL-1 gene involved in acute leukemia. *Cancer Res* **54**: 2326–2330

Gustafson CE, Young J, Leggett B, Searle J and Chenevix-Trench G (1994) Loss of heterozygosity on the long arm of chromosome 11 in colorectal tumours. *Br J Cancer* **70**: 395–397

Hampton GM, Penny LA, Baergen RN, Larson A, Brewer C, Liao S, Busby-Earle RMC, Williams AWR, Steel CM, Bird CC, Stanbridge EJ and Evans GA (1994). Loss of heterozygosity in cervical carcinoma: Subchromosomal localization of a putative tumour-suppressor gene to chromosome 11q22–q24. *Proc Natl Acad Sci USA* **91**: 6953–6957

 *© 2000 Cancer Research Campaign*

Herbst RA, Larson A, Weiss J, Cavenee WK, Hampton GM and Arden KC (1995) A defined region of loss of heterozygosity at 11q23 in cutaneous malignant melanoma. *Cancer Res* **55**: 2494–2496

Hui ABY, Lo KW, Leung SF, Choi PHK, Fong Y, Lee JCK and Huang DP (1996) Loss of heterozygosity on the long arm of chromosome 11 in nasopharyngeal carcinoma. *Cancer Res* **56**: 3225–3229

Iselius L, Lindsten J, Aurias A, Fraccaro M, Bastard C, Bottelli AM, Bui TH, Caufin D, Dalpra L, Delendi N, et al (1983) The 11q;22q translocation: a collaborative study of 20 new cases and analysis of 110 families. *Hum Genet* **64**: 343–355

Keldysh PL, Dragani TA, Fleischman EW, Konstantinova LN, Perevoschikov AG, Pierotti MA, Della Porta G and Kopnin BP (1993) 11q deletions in human colorectal carcinomas: cytogenetics and restriction fragment length polymorphism analysis. *Genes, Chromosomes & Cancer* **6**: 45–50

Konstantinova LN, Fleischman EW, Knisch VI, Perevozchikov AG and Kopnin BP (1991) Karyotype pecularities of human colorectal adenocarcinomas. *Hum Genet* **86**: 491–496

Koreth J, Bakkenist CJ and McGee JO'D (1997) Allelic deletions at chromosome 11q22–q23.1 and 11q25–qterm are frequent in sporadic breast but not colorectal cancers. *Oncogene* **14**: 431–437

Lindblom A, Sandelin K, Iselius L, Dumanski J, White I, Nordenskjold M and Larsson C (1994) Preidsposition for breast cancer in carriers of constitutional translocation 11q;22q. *Am J Hum Genet* **54**: 871–876

Monaco C, Negrini M, Sozzi G, Veronese ML, Vorechovsky I, Godwin AK and Croce CM (1997) Molecular cloning and characterization of LOH11CR2A, a new gene within a refined minimal region of LOH at 11q23. *Genomics* **46**: 217–222

Negrini M, Rasio D, Hampton GM, Sabbioni S, Rattan S, Carter SL, Rosenberg AL, Schwartz GF, Shiloh Y, Cavenee WK and Croce CM (1995) Definition and refinement of chromosome 11 regions of loss of heterozygosity in breast cancer: identification of a new region at 11q23.3. *Cancer Res* **55**: 3003–3007

Rasio D, Negrini N, Manenti G, Dragani TA and Croce CM (1995) Loss of heterozygosity at chromosome 11q in lung adenocarcinoma: Identification of three independent regions. *Cancer Res* **55**: 3988–3991

Risio M, Reato G, di Celle PF, Fizzotti M, Rossini FP and Foa R (1996) Microsatellite instability is associated with the histological features of the tumour in nonfamilial colorectal cancer. *Cancer Res* **56**: 5470–5474

Robertson G, Coleman A and Lugo TG (1996) A malignant melanoma tumour suppressor on human chromosome 11. *Cancer Res* **56**: 4487–4492

Tomlinson IPM and Bodmer WF (1996) Chromosome 11q in sporadic colorectal carcinoma: patterns of allele loss and their significance for tumorigenesis. *J Clin Pathol* **49**: 386–390

Tomlinson IPM, Gammack AJ, Stickland JE, Mann GJ, Mackie RM, Kefford RF and McGee JO'D (1993) Loss of heterozygosity in malignant melanoma at loci on chromosomes 11 and 17 implicated in the pathogenesis of other cancers. *Genes, Chromosomes & Cancer* **7**: 169–172

Tomlinson IPM, Stickland JE, Lee ASG, Bromley L, Evans MF, Morton J and McGee JO'D (1995) Loss of heterozygosity on chromosome 11q in breast cancer. *J Clin Pathol* **48**: 424–428

Wang SS, Esplin ED, Li JL, Huang L, Gazdar A, Minna J and Evans GA (1998) Alterations of the PPP2R1B gene in human lung and colon cancer. *Science* **282**: 284–287

Wingo PA, Ries LAG, Rosenberg HM, Miller DS and Edwards BK (1998) Cancer incidence and mortality, 1973–1995. *Cancer* **82**: 1197–1207

*© 2000 Cancer Research Campaign*
